# Stroke Prevention in Atrial Fibrillation: Latest Clinical Trials and Guidelines

**DOI:** 10.3390/ph5040384

**Published:** 2012-04-05

**Authors:** Luciana Armaganijan, Dimpi Patel, Cristiano Dietrich, Carlos A. Morillo

**Affiliations:** 1 Cardiac Arrhythmias and Electrophysiology, Dante Pazzanese Institute of Cardiology, Sao Paulo 04012-180, Brazil; 2 1506 Bristol Court, Elizabethtown, KY 42701, USA; 3 Department of Medicine, Cardiology Division, Electrophysiology Service, Paulista School of Medicine, UNIFESP, Sao Paulo 04024-002, Brazil; 4 Department of Medicine, Cardiology Division, Arrhythmia Service, McMaster University, Population Health Research Institute, Hamilton, ON L8L 2X2, Canada

**Keywords:** atrial fibrillation, anticoagulants, stroke, prevention

## Abstract

Atrial Fibrillation (AF) is the most common sustained arrhythmia and 1/6 strokes is attributed to AF. The cornerstone of treatment remains maintaining sinus rhythm or appropriate ventricular rate control in addition to prevention of stroke. Oral anticoagulation therapy (OAC) with vitamin K antagonists (VKAs) has been the gold standard for almost 50 years and a significant reduction in the risk of stroke in patients with AF has been demonstrated. Nonetheless, only 50% of patients with guideline recommendations for OAC treatment actually receive VKAs and half of these will discontinue therapy within 3 to 5 years with only another half achieving therapeutic ranges more than 50% of the time. The aforementioned limitations in addition with frequent blood monitoring have prompted the development of a series of new OAC therapies. The present review focuses on the current pharmacological management for stroke prevention in patients with AF based on current and emerging evidence.

## 1. Introduction

Atrial Fibrillation (AF) is the most common sustained arrhythmia. It affects 1–2% of the general population and is associated with an increased risk of stroke, and several studies suggest that 1/6 strokes may be attributed to AF [1,2]. Currently, antiarrhythmic, and rate control medications are the mainstay therapy in addition with oral anticoagulation (OAC) therapy for the prevention of stroke.

The primary goals of the pharmacological management of AF are fourfold: (1) Symptom relief (*i.e*., rate or rhythm control strategies), (2) Control of risk factors that promote and facilitate AF (*i.e*., upstream therapy, ACE inhibitors, aldosterone receptor blockers, statins, *etc*.), (3) Stroke and systemic embolism prevention, and (4) Reduction in mortality and morbidity associated with AF. The present review focuses on the current pharmacological management for stroke and systemic embolism prevention based on current and emerging evidence derived from large randomized clinical trials.

### 1.1. Stroke and Systemic Embolism Risk Stratification

AF is associated with a fivefold increased risk of systemic embolism or stroke with an absolute risk ranging from less than 1% to 20% per year, depending on patient age and presence of additional risk factors [3]. Several risk stratification scores have been devised in an attempt to facilitate the identification and provide an overall estimate of the risk of stroke in patients with AF. The CHADS_2_ score is among the most widely used and validated risk scores and is based on a point system in which 2 points are assigned for a history of stroke or transient ischemic attack (TIA) and 1 point is assigned for any of the following risk factors: congestive heart failure, systemic hypertension, age equal or greater than 75 years and diabetes mellitus. The annual risk of stroke increases as the risk score is higher, providing guidance for the use of oral anticoagulants (OACs) in patients with AF (Table 1) [4].

**Table 1:** CHADS_2_ score and annual adjusted stroke rate.

CHADS_2_ Score	Adjusted Stroke Rate (%/year) (95% CI)
0	1.9 (1.2–3.0)
1	2.8 (2.0–3.8)
2	4.0 (3.1–5.1)
3	5.9 (4.6–7.3)
4	8.5 (6.3–11.1)
5	12.5 (8.2–17.5)
6	18.2 (10.5–27.4)

In addition to left ventricular systolic dysfunction, recent reports have suggested that the presence of left atrial thrombus, complex aortic plaques, spontaneous echo-contrast and low left atrial appendage velocities (≤20 cm/s; RR 1.7; *p* < 0.01) are independent predictors of stroke and thromboembolism [5]. Nonetheless, none of the above has been introduced as part of any of the risk stratification scores. More recently, the ESC guidelines incorporated the CHA_2_DS_2_-VASc score, in which 1 point is assigned to congestive heart failure, hypertension, age between 65 and 74 years, diabetes mellitus, vascular disease (myocardial infarction, complex aortic plaque, and peripheral artery disease (PAD), including prior revascularization, amputation due to PAD, or angiographic evidence of PAD, *etc*.), and female gender and 2 points are assigned for a history of stroke or TIA, or age ≥75 (Table 2). The annual adjusted stroke risk according to the CHA_2_DS_2_-VASc score is summarized in Table 3. This risk stratification scheme is more complex; however, a recent study indicates that the CHADS-VASc c-statistic is very similar to that for CHADS2, but the CHADS-VASc improves risk prediction among patients at lower risk of stroke, *i.e*., with CHADS2 score ≤1 [6].

The Canadian Cardiovascular Society (CCS) AF Guidelines and the American Guidelines continue to recommend the use of the CHADS2 score and suggest the use of the more complex CHA_2_DS_2_-VASc to further fine tune risk stratification on low or intermediate risk patients. All current guidelines recommend the routine use of either risk stratification score in all patients with AF as a mean to identify patients at higher risk of stroke and systemic embolism and, thereby, implement appropriate antithrombotic therapy [7].

**Table 2:** CHA_2_DS_2_-VASc score.

Risk Factor	Score
Congestive heart failure/LVEF <40%	1
Systemic hypertension	1
Age ≥75 years	2
Diabetes mellitus	1
Stroke/TIA/thromboembolism	2
Vascular disease *	1
Age 65–74 years	1
Female	1

* Myocardial infarction, complex aortic plaque and peripheral artery disease (PAD), including prior revascularization, amputation due to PAD, or angiographic evidence of PAD, *etc*. TIA: transient ischemic attack.

**Table 3:** Annual adjusted stroke rate according to the CHA_2_DS_2_-VASc score.

CHA_2_DS_2_-VASc Score	Adjusted Stroke Rate (%/year)
0	0
1	1.3%
2	2.2%
3	3.2%
4	4.0%
5	6.7%
6	9.8%
7	9.6%
8	6.7%
9	15.2%

Current ESC guidelines recommend OAC therapy in patients with a CHADS_2_ score ≥2, targeting international normalized ratio (INR) values between 2.0 and 3.0. In patients with a CHADS_2_ score of 1, either aspirin (75–325 mg daily) or warfarin is recommended, depending on a detailed stroke risk assessment. In the absence of stroke risk factors (e.g., CHADS_2_ of 0), either aspirin 75–325 mg daily or no antithrombotic therapy is recommended. CHA_2_DS_2_-VASc score is recommended to be used for further risk stratification in patients with a CHADS_2_ score of 0 or 1; and the recommendation on anticoagulation or aspirin is made based on this assessment [5].

The ACCF/AHA/HRS guidelines recommend chronic OAC therapy to achieve an INR of 2.0–3.0 in patients at high risk of stroke, unless contraindicated. A lower target INR of 2.0 (range 1.6 to 2.5) may be considered in patients over 75 years old considered at increased risk of bleeding complications but without frank contraindications to oral anticoagulant therapy [8].

The CCS-AF guidelines suggest that all patients with AF or atrial flutter (paroxysmal, persistent or permanent) should be stratified using the CHADS_2_ score and that most patients should receive antithrombotic therapy. Patients at very low risk of stroke (CHADS_2_ = 0) are recommended to receive aspirin (81–325 mg/day). In the absence of standard risk factors for stroke, antithrombotic therapy may not be required. Patients at low risk of stroke (CHADS_2_ = 1) should receive either aspirin or OAC therapy (warfarin or dabigatran) depending on individual risk/benefit considerations. For patients at moderate risk of stroke (CHADS_2_ ≥ 2) OAC therapy (either warfarin [INR 2.0–3.0] or dabigatran) is indicated with preference to dabigatran, at the dose of 150 mg twice daily [7]. It is likely that with the advent and further familiarization with newer OACs that have a better safety profile than VKAs more patients in the low-intermediate risk will be prescribed with these newer agents.

### 1.2. Bleeding Risk

Bleeding is the single most important factor that limits widespread indication for OAC therapy. Although recent reports indicate low rates of OAC related intracerebral hemorrhage (between 0.1 and 0.6%), major bleeding can occur in up to 4% per year making bleeding risk assessment crucial before initiating OAC therapy. In order to avoid bleeding, careful dose titration and adequate hypertension control are the cornerstone for the prevention of bleeding.

Stroke risk factors are also associated with and a higher risk for hemorrhage, *i.e*., greater bleeding risk is associated with increasing CHADS_2_ score. A variety of scoring systems have been developed to identify clinical risk factors associated with an incremental risk for hemorrhage. The HAS-BLED score offers useful predictive capacity for bleeding. One point is assigned to each of the following markers: hypertension, abnormal renal/liver function, stroke, bleeding history or predisposition, labile INR, elderly and concomitant use of drugs/alcohol (Table 4).

**Table 4:** HAS-BLED score.

Letter	Characteristics	Points
H	Hypertension	1
A	Abnormal renal and liver function (1 point each)	1 or 2
S	Stroke	1
B	Bleeding	1
L	Labile INR	1
E	Elderly (≥65 years old)	1
D	Drugs or alcohol 1 point each)	1 or 2

Hypertension is defined as systolic blood pressure >160 mmHg, abnormal kidney function is defined as the presence of chronic dialysis or renal transplantation or serum creatinine ≥200 µmol/L and abnormal liver function is defined as chronic hepatic disease (e.g., cirrhosis) or biochemical evidence of significant hepatic derangement (bilirubin >2 × upper limit of normal in association with AST/ALT >3 × upper limit normal). Drugs users refer to concomitant use of drugs such as antiplatelet agents, non-steroidal anti-inflammatory drugs, *etc*. A score ≥3 indicates “high risk”, requiring caution and regular review following the initiation of antithrombotic therapy [9].

### 1.3. Antithrombotic Therapy

Vitamin K antagonists (VKAs; e.g., warfarin, acenocuomarol) remain the standard of care for OAC in the prevention of stroke in AF. Aspirin is also an alternative but associated with lower relative risk reduction in stroke or systemic embolism. Adjusted dose warfarin and aspirin reduce the incidence of stroke by 64% and 22%, respectively; with warfarin being significantly superior to ASA [10]. Nonetheless, OACs, in spite of a clear indication, are underused in approximately 50% of AF patients [11,12,13,14]. Similarly, despite frequent INR measurement, less than 50% maintain an INR within therapeutic ranges [15]. Finally, 50% of patients discontinue warfarin after 3 to 5 years, due to poor INR control, bleeding or other complications [14]. The aforementioned limitations of VKAs have stimulated the development of new OACs expected to overcome most of warfarin limitations. Alternatives to warfarin include antiplatelet therapy, new OACs and exclusion of the left atrial appendage.

## 2. Combined Anti-Platelets Strategy

Two trials have evaluated the effectiveness and safety of the combination of aspirin and clopidogrel for the prevention of stroke in patients with AF. The Atrial Fibrillation Clopidogrel Trial with Irbesartan for Prevention of Vascular Events (ACTIVE W) trial [16] was designed to compare the effects of warfarin (target INR of 2.0–3.0) *vs*. the combination of clopidogrel (75 mg/day) plus aspirin (75 to 100 mg/day) in AF patients with at least one additional risk factor for stroke. The main impact was derived from a significant reduction of stroke (RR 1.72; 95% CI, 1.24 to 2.37; *p* = 0.001) and systemic embolism (RR 4.66; 95% CI, 1.58 to 13.8; *p* = 0.005) related to warfarin use. There were no differences in the occurrence of major bleeding between groups (2.42% per year with clopidogrel plus aspirin *vs*. 2.21% per year with warfarin, RR 1.1; 95% CI 0.83 to 1.45; *p* = 0.53) [16].

The ACTIVE A trial included patients who were unsuitable for therapy with OACs and was designed to compare the effects of the combination of clopidogrel (75 mg daily) and aspirin (75 to 100 mg daily) to aspirin alone on the prevention of stroke and cardiovascular events (non-central nervous system embolism, MI or vascular death) [17]. A total of 7,554 AF patients with at least one additional risk factor for vascular events were enrolled in the study. The combined therapy was superior to aspirin alone with an 11% relative risk reduction on the primary outcome, which was primarily driven by a 28% reduction in the occurrence of stroke (RR 0.72; 95% CI, 0.62 to 0.83; *p* < 0.001). The combination of clopidogrel and aspirin, however, was associated with higher risk of intracranial and extra cranial bleeding, from 1.3% to 2.0% per year (RR 1.57; 95% CI, 1.29 to 1.92; *p* < 0.001) [17].

The ACTIVE trials indicate that the combination of clopidogrel and aspirin is more effective but with a higher bleeding risk than aspirin alone, in patients unsuitable for OACs. Warfarin is clearly superior to the combination of aspirin and clopidogrel for the prevention of vascular events such as stroke and systemic embolism in patients with AF. With the advent of newer OACs the role for clopidogrel + ASA for stroke prevention in AF patients seems limited.

Current ESC guidelines recommend maintaining an INR in the range of 3.0–3.5 rather than adding aspirin in those patients who develop ischemic stroke despite having therapeutic INR’s, with the caveat that increased bleeding including intracranial hemorrhage, may overcome the benefits of OACs [5].

## 3. New Anticoagulants

### 3.1. Thrombin Inhibitors

#### 3.1.1. Ximelagatran

The prodrug ximelagatran, a direct thrombin inhibitor, is converted to the active agent melagatran in the liver and other tissues through dealkylation and dehydroxylation and it is rapidly absorbed by the small intestine. Ximelagatran is taken orally twice daily. The Stroke Prevention using ORal Thrombin Inhibitor in atrial Fibrillation III and IV (SPORTIF III and V) trials were designed to compare dose-adjusted warfarin (target INR 2.0 to 3.0) to ximelagatran (36 mg twice daily) [18,19]. The studies were similar except for the open-label with blinded event assessment design of the SPORTIF III and the double-blind design of the SPORTIF V. In the SPORTIF III, the occurrence of the primary endpoint (stroke or systemic embolism) and combined minor and major hemorrhages were lower in the ximelagatran group compared to warfarin (1.6% per year *vs*. 2.3% per year, absolute RR of 0.7%; 95% CI, 0.1 to 1.4, RRR 29%, 95% CI, −6.5 to 52; and 29.8% *vs.* 25.8% per year; RRR of 14%; 95% CI, 4 to 22; *p* = 0.007). No differences on the rates of disabling or fatal stroke, mortality and major bleeding were seen between the groups (6.1% per year *vs*. 4.6% per year in the warfarin and ximelagatran groups, RRR of 25%, *p* = 0.022) [18]. The SPORTIF V also showed promising results with lower risk of stroke and thromboembolic events (1.6% per year *vs*. 1.2% per year with warfarin) (absolute difference 0.45% per year; 95% CI, −0.13 to 1.03; *p* < 0.001) as well as lower combined major and minor bleeding events (37% *vs*. 47% per year; 95% CI, −14% to −6%, *p* < 0.001) with ximelagatran with similar rates of stroke, major bleeding and mortality to warfarin [19]. These studies indicated that ximelagatran was non-inferior to warfarin and had a better safety profile. Unfortunately, ximelagatran was withdrawn due to significant liver toxicity. The results of these trials, however, opened a new perspective of the effectiveness of another unmonitored oral anticoagulant therapy for stroke prevention in AF patients.

#### 3.1.2. Dabigatran Etexilate

Dabigatran etexilate, a prodrug of dabigatran, which reversibly inhibits the active site of thrombin, is rapidly and completely converted to dabigatran by esterases and reaches peak plasma levels about 2 h after oral administration. It has as an oral bioavailability of about 6% and a half-life of 12 to 14 h requiring twice-daily administration. Approximately 80% of the drug is excreted unchanged by the kidneys [20].

The Randomized Evaluation of Long-Term Anticoagulant Therapy (RELY) [21] was a randomized clinical trial that used a non-inferiority study design to assess the safety and efficacy of dabigatran etexilate in 18,113 non-valvular AF patients with at least one additional risk factor for stroke. Two doses were tested: 110 mg BID and 150 mg BID. Dabigatran 150 mg BID was superior to warfarin in reducing the incidence of composite of stroke (including hemorrhagic) or systemic embolism by 34% (RRR 0.66; 95% CI, 0.53–0.82; *p* < 0.001) with no significant difference in the occurrence of major bleeding (3.36% per year in the group that received warfarin and 3.11% per year in the group receiving dabigatran 150 mg, *p* = 0.31). Dabigatran 110 mg BID was non-inferior to warfarin for the outcome of the composite of stroke or systemic embolism and associated with a 20% RRR in major bleeding when compared to warfarin. Interestingly, the occurrence of intracerebral hemorrhage was significantly lower with both dabigatran doses compared to warfarin (RRR 74%; *p* < 0.001 and RRR 69%; *p* < 0.001, for 150 mg BID and 110 mg BID, respectively). The mortality rate was 4.13% per year in the warfarin group, as compared with 3.75% per year with 110 mg of dabigatran (*p* = 0.13) and 3.64% per year with 150 mg of dabigatran (*p* = 0.051). In contrast to ximelagatran, dabigatran was not associated with liver toxicity. The most frequent adverse event in both dabigatran groups was dyspepsia (11.8% patients with 110 mg, 11.3% patients with 150 mg compared to 5.8% with warfarin, *p* < 0.001 for both), leading to drug discontinuation in the minority of patients (2.2% and 2.1% of patients in the dabigatran 110 mg and 150 mg arm, respectively and 0.6% of patients in the warfarin arm) [21].

The RELY trial provides a strong new alternative to VKAs with the same efficacy and better safety profile at the low dose and clear superiority with no increased risk of bleeding compared to warfarin at the high dose. The CCS and ESC guidelines suggest that dabigatran may be considered as an alternative to adjusted dose VKA therapy. In cases of low risk of bleeding (e.g., HAS-BLED score of 0–2), dabigatran 150 mg twice daily may be considered, in view of the improved efficacy in the prevention of stroke and systemic embolism but lower rates of intracranial hemorrhage and similar rates of major bleeding events compared with warfarin; in cases of measurable risk of bleeding (e.g., HAS-BLED score of ≥3), dabigatran 110 mg twice daily is preferable, in view of a similar efficacy in the prevention of stroke and systemic embolism but lower rates of intracranial hemorrhage and of major bleeding compared with VKA and (probably) aspirin [5].

A sub analysis of the RELY study showed that higher CHADS_2_ scores were associated with increased risks for stroke or systemic embolism, bleeding, and death in patients with AF receiving oral anticoagulants [22]. Dabigatran is a reasonable alternative to warfarin in patients requiring cardioversion. A post hoc analysis showed that the rate of thromboembolism and major bleeding within 30 days of cardioversion on the 2 doses of dabigatran were low and comparable to those on warfarin with or without transesophageal echocardiography guidance [23]. In regards to age, both doses of dabigatran compared with warfarin have lower risks of both intracranial and extracranial bleeding in patients aged <75 years. In those aged ≥75 years, intracranial bleeding risk is lower but extracranial bleeding risk is similar or higher with both doses of dabigatran compared with warfarin [24].

Dabigatran is now approved in North America for stroke prevention in patients with nonvalvular AF. Documentation of creatinine clearance is necessary prior to initiation of dabigatran. A dose of 150 mg twice daily is recommended in patients with creatinine clearance >30 mL/min, whereas patients with creatinine clearance between 15 and 30 mL/min should be treated with 75 mg twice daily. Dabigatran is contraindicated in patients with a creatinine clearance <15 mL/min or patients on dialysis. It is important to note that the FDA approved the 150 mg/bid dose and 75 mg/bid for patients with renal impairment, however, this is not the recommendation elsewhere. The CCS-AF guidelines recommend for patients at moderate and high risk preference to dabigatran rather than warfarin based on the risk benefit profile with non-inferior/superior efficacy and a better bleeding profile at the low dose (110 mg/bid) approved for prescription in Canada [7].

### 3.2. Factor Xa Inhibitors

#### 3.2.1. Indirect Factor Xa Inhibitors

Idraparinux: Idraparinux is a subcutaneously administered indirect factor Xa inhibitor with a long half-life of 80 h which allows weekly administration. The Atrial fibrillation trial of Monitored, Adjusted Dose vitamin K antagonist, comparing Efficacy and safety with Unadjusted SanOrg 34006/idraparinux (AMADEUS) study was a multicenter, randomized, open-label non-inferiority trial that evaluated the effects of idraparinux in AF patients with at least one additional risk factor for stroke [25]. 4,576 individuals were recruited. Subcutaneous idraparinux (2.5 mg once weekly, adjusted for renal function) was compared to oral VKA. Idraparinux was non-inferior to warfarin, however, the incidence of clinically significant major bleeding was higher in the idraparinux group (19.7% *vs.* 11.3% per year for idraparinux and warfarin, respectively, *p* < 0.0001), resulting in early trial termination. Furthermore, a higher incidence of intracranial bleeding was also observed in the idraparinux arm (1.1% per year *vs*. 0.4% per year, respectively; RR 2.58; 95% CI, 1.18 to 5.63; *p* = 0.014) [25]. These results led to the introduction of a structurally similar formulation of idraparinux with the exception that it contains a biotinylated segment which has a strong and specific affinity for avidin, a neutralizing agent. Administration of avidin allows rapid neutralization of the anticoagulant activity of the drug. Biotinylated idraparinux (idrabiotaparinux) was compared to warfarin in the phase III Evaluation of Weekly Subcutaneous Biotinylated Idraparinux *vs*. Oral Adjusted-dose Warfarin to Prevent Stroke and Systemic Thromboembolic Events in Patients With Atrial Fibrillation (BOREALIS-AF) study. The study was prematurely terminated, and the reason remains unclear [26].

#### 3.2.2. Direct Factor Xa Inhibitors

##### 3.2.2.1. Rivaroxaban

Rivaroxaban is a direct Xa factor inhibitor with high bioavailability, rapid onset of action (peak plasma concentration 2.5 to 4 h after dosing) and a half-life of 7 to 13 h. Approximately one third is renally excreted. The Rivaroxaban Once Daily oral Direct factor Xa inhibition Compared with Vitamin K Antagonism for Prevention of Stroke and Embolism Trial in Atrial Fibrillation study (ROCKET-AF) was a randomized, double-blind, double-dummy, event-driven trial, which aimed to establish the non-inferiority of rivaroxaban compared with warfarin in patients with non-valvular AF who have a history of stroke or at least 2 additional independent risk factors for future stroke. Patients were randomly assigned to receive 20 mg rivaroxaban once a day (with dose adjustment for renal function) or dose-adjusted warfarin (targeting INR values between 2.0 and 3.0). The primary efficacy end point was a composite of all-cause stroke and non-central nervous system systemic embolism and the primary safety end point was the composite of major and clinically relevant non-major bleeding events; 14,264 patients from 1,178 sites in 45 countries were enrolled in the study. In the on-treatment analysis, rivaroxaban was associated with a 21% relative risk reduction in the occurrence of stroke and non-CNS systemic embolism (1.7% *vs*. 2.2%, respectively, *p* = 0.015). Additionally, in the intention to treat analysis rivaroxaban showed comparable benefits to warfarin (2.1% *vs*. 2.4%, *p* < 0.001 for non-inferiority). Rivaroxaban was not associated with an increase in major and non-major clinically relevant bleeding events compared to warfarin (14.9% *vs*. 14.5%, *p* = 0.442). The drug was well tolerated in the study, and rates of discontinuation due to adverse events were similar to those seen for patients on warfarin [27]. As opposed to the PROBE design of the RELY, the ROCKET-AF had a double dummy design which could have explained the observed results as the TTR was only 55%. It’s important to note, however, that there is no evidence that either design is superior or that the PROBE design introduces any significant bias to the results.

In conclusion, rivaroxaban was found to be non-inferior to warfarin but no superiority was clearly demonstrated. In view of the safety profile (less intracranial and fatal bleedings when compared to warfarin), rivaroxaban may be an alternative in patients at moderate and high risk, considering the limitations of warfarin use. Close evaluation is recommended in patients with moderate renal impairment (creatinine clearance between 30 and 50 mL/min). Rivaroxaban should be avoided in patients with severe renal impairment (creatinine clearance <30 mL/min). In cases of acute renal failure, the treatment should be discontinued.

##### 3.2.2.2. Apixaban

Apixaban is administrated twice daily and has a half-life of 12 h. Approximately 25% is excreted by the kidneys. Apixaban was tested in two large randomized clinical trials for stroke prevention in AF patients: AVERROES and ARISTOTLE. The Apixaban *Versus* Acetylsalicylic Acid to Prevent Strokes (AVERROES) was a randomized double-blind study that compared the efficacy of apixaban with aspirin for the prevention of stroke or systemic embolism in AF patients considered unsuitable for VKA treatment. Patients were randomized to apixaban 5 mg twice daily or aspirin (81 to 325 mg once daily) [27]. The study was terminated early due to significant differences in the primary end point (stroke or non-central systemic embolism) favoring the apixaban arm (hazard ratio with apixaban, 0.45; 95% CI 0.32 to 0.62, *p* < 0.001). The rates of death and major bleeding did not differ between the groups (hazard ratio, 0.79; 95% CI 0.62 to 1.02, *p* = 0.07 and 1.13; 95% CI, 0.74 to 1.75, *p* = 0.57, respectively. There were 11 cases of intracranial bleeding with apixaban and 13 with aspirin. The risk of a first hospitalization for cardiovascular causes was reduced with apixaban as compared with aspirin (12.6% per year *vs*. 15.9% per year, *p* < 0.001) [28].

Apixaban was compared with warfarin in the Apixaban for Reduction In Stroke and Other ThromboemboLic Events in Atrial Fibrillation study (ARISTOTLE), a phase III randomized, double-blind double-dummy trial for the prevention of stroke and systemic embolism in subjects with non-valvular atrial fibrillation and at least one additional risk factor for stroke [29]. The primary outcome of the study was stroke or systemic embolism. Combined ischemic stroke, hemorrhagic stroke, systemic embolism and all cause death were the secondary outcomes. 18,201 patients were included. Compared with warfarin, apixaban reduced the primary outcome by 21% (*p* = 0.01), major bleeding by 31% (*p* < 0.001), and all-cause mortality by 11% (*p* = 0.047). Apixaban was well tolerated and resulted in fewer drug discontinuation compared to warfarin [28]. The results of the ARISTOTLE trial showed important advantages of apixaban over warfarin, including lower rates of stroke, major bleeding, and mortality, and thus may be preferred over warfarin in selected patients.

##### 3.2.2.3. Edoxaban

The Effective aNticoaGulation with factor xA Next GEneration in Atrial Fibrillation trial (ENGAGE-TIMI 48) is a multicenter, double-blind randomized trial that is comparing the efficacy of two different doses of edoxaban (30 or 60 mg once-daily) with warfarin on the occurrence of stroke and thromboembolic systemic events in approximately 16,500 AF patients. The results are expected in 2012 [30].

Table 5 and Table 6 show the distinguishing features of the new oral anticoagulants and trials. The completed randomized trials of new anticoagulants for prevention of stroke in patients with AF and the summary of the results are represented in Table 7 and Table 8, respectively.

**Table 5:** Distinguishing features of the new oral anticoagulants.

Feature	Dabigatran	Rivaroxaban	Apixaban
Doses	110 mg, 150 mg	20 mg (15 mg)	5 mg (2.5 mg)
Dose frequency	Twice-daily	Once-daily	Twice-daily
Half life	12–14 h	7–13 h	12 h
Renal excretion	80%	33%	25%

**Table 6:** Distinguishing features of the studies on new oral anticoagulants for stroke prevention in AF patients.

Feature	RELY	ROCKET	ARISTOTLE
N	18,113	14,266	18,206
Mean CHADS_2_ score	2.1	3.5	2.1
Design	Probe	Double-blind	Double-blind
TTR (%) *	64	55	62
% VKA naive	50	38	43

* Time within the therapeutic range for warfarin.

**Table 7:** Completed randomized trials of new anticoagulants for stroke prevention in patients with AF.

Agent	Trial	Conclusion
**Ximelagatran**	SPORTIF III [18] SPORTIF V [19]	Non-inferior to warfarin ^†^ Major bleeding—Similar between groups Withdrawn due to hepatotoxicity
**Dabigatran**	RELY [21]	Dabigatran 150mg—Superior to warfarin ^†^ Dabigatran 110mg—Non-inferior to warfarin ^†^ Major bleeding-lower with 110 mg of dabigatran Reduction in vascular mortality with dabigatran 150 mg
**Idraparinux**	AMADEUS [25]	Non-inferior to warfarin ^†^ Early termination due to significantly more clinically relevant bleeding with idraparinux
**Rivaroxaban**	ROCKET-AF [26]	Non-inferior to warfarin ^†^ Comparable benefits to warfarin for non-inferiority in the intent to treat population No differences in major and non-major clinically relevant bleeding events
**Apixaban**	AVERROES [27]	Superior to warfarin ^†^ No differences in rates of death major and intracranial bleeding between the groups Risk of a first hospitalization for cardiovascular causes reduced with apixaban
	ARISTOTLE [28]	Superior to warfarin * Well tolerated Fewer drug discontinuation

^†^ For prevention of thromboembolic event includes stroke and systemic embolism; * reduced stroke, systemic embolism, major bleeding, and mortality.

**Table 8:** Summary of results.

Feature	Dabigatran 150 mg bid	Rivaroxaban 20 mg once daily	Apixaban 5 mg bid
Stroke	−36%	−12%	−21%
Ischemic stroke	−24%	−1%	−8%
ICH	−74%	−33%	−49%
Death	−12%	−8%	−11%
Bleeding	−7%	+3%	−31%
GI Bleeding	+36%	~40%	−11%
Other	Dyspepsia	-	-

ICH: intracranial hemorrhage; GI: gastrointestinal.

## 4. Bleeding Management

A major concern with the safety of the new drugs has been the lack of reversal agents in case of serious bleeding or in case of an emergency intervention requiring immediate correction of coagulation. Recently, in a randomized, double-blind, placebo-controlled study, the use of prothrombin complex concentrate (PCC) was shown to effectively reverse the anticoagulant effects of rivaroxaban but not of dabigatran. Measurements of standard laboratory markers of anticoagulation were used as evidence of reversal. Rivaroxaban prolonged the activated prothrombin time, which was immediately reversed by PCC. Similar results were seen when the endogenous thrombin potential was used as a measure of anticoagulant effect. Whether PCC actually will stop rivaroxaban-induced bleeding was not demonstrated. PCC was not effective at reversing the anticoagulant effects of dabigatran.

The new anticoagulants have an important feature that leads to reversibility, the relatively short half-life. Importantly, despite the lack of a specific antidote, most serious types of bleeding and intracranial hemorrhage were reduced with dabigatran and apixaban as compared to warfarin. Rivaroxaban showed no differences in major and non-major clinically relevant bleeding events as compared to warfarin [31].

## 5. Conclusions

The management of AF has significantly changed in the past decade and stroke prevention remains a challenge. Despite the proven efficacy of VKA, its limitations have stimulated the development of new oral anticoagulants expected to overcome most of warfarin disadvantages. Several newer oral anticoagulant agents with better safety profile and ease of administration that do not need routine monitoring will change and improve as well as expand the use of OACs in patients with AF at risk of stroke. Different agents such as dabigatran and apixaban have shown better safety as well as improved outcomes, the ability to tailor treatment based on the different characteristics of the new OACs will significantly reduce stroke and improve overall outcomes in the prevention of stroke in patients with AF.
